# Does the social context of early alcohol use affect alcohol-related harms in adulthood? Findings from a national birth cohort

**DOI:** 10.1016/j.ypmed.2019.105947

**Published:** 2020-01

**Authors:** James White, Steven Bell, G. David Batty

**Affiliations:** a Centre for Trials Research, Cardiff University, 4th floor, Neuadd Meirionnydd, Heath Park, Cardiff CF14 4YS, UK; b The National Institute for Health Research Blood and Transplant Unit in Donor Health and Genomics at the University of Cambridge, University of Cambridge, Cambridge CB1 8RN, UK; c British Heart Foundation Cardiovascular Epidemiology Unit, University of Cambridge, Cambridge CB1 8RN, UK.; d Stroke Research Group, Department of Clinical Neurosciences, University of Cambridge, Cambridge, CB2 0QQ, UK; e Department of Epidemiology and Public Health, University College London, London WC1E 7HB, UK; f School of Biological and Population Health Sciences, Oregon State University, Corvallis, OR, USA

**Keywords:** Alcohol, Parents, Home, Adolescents

## Abstract

Internationally, laws on the provision of alcohol commonly exempt that provided by parents and/or consumed in private premises. Whether these exemptions mitigate alcohol-related harms, as has been posited, is unclear. We used data from 10,968 individuals (5216 women) from the 1970 British Birth Cohort Study. Exposures, self-reported at 16-years of age, were consumption of alcohol with specific people (including parents, siblings and friends) and acquisition from different places (including their own home). The outcomes, self-reported at 30-years of age, were high alcohol consumption (>14 units of alcohol in the last week), and screening positive for a possible alcohol problem using the cutting down, being annoyed by criticism, feeling guilty, and eye-openers (CAGE) questionnaire. At 30-years of age, 32.1% of study members consumed >14 units in the last week and 14.3% screened positive on the CAGE questionnaire. Neither consuming alcohol with parents nor the acquisition of alcohol from home was associated with later high consumption or alcohol problems. There was a suggestion, however, that drinking with other teenagers was related to an increased risk of both outcomes (consumption: 1.32 (1.16, 1.51); alcohol problems: 1.27 (1.01, 1.58), as was acquisition from an off-license (consumption: 1.23 (0.99, 1.51); alcohol problems: 1.49 (1.17, 1.90). This study strengthens the evidence that alcohol consumption with parents, or acquisition from home, does not protect against later alcohol-related harms.

## Highlights

• Alcohol laws commonly exempt that provided by parents and/or consumed in private premises.
• Drinking with parents did not protect against later alcohol problems.
• Getting alcohol from home did not protect against later alcohol problems.
• This study adds to evidence questioning exemptions allowing underage drinking.

## 1 Introduction

Alcohol use, particularly at high levels of consumption, is associated with a series of adverse social, mental, and behavioural disorders (Rehm et al., 2009). In many countries, legislation allows children to consume alcohol at home (Steffens and Sarrazin, 2016) and parents are a frequent provider before the legal age of purchase (NHS Digital, 2017). For example, in the United Kingdom, although alcohol cannot be sold to anyone under the age of 18, it can be consumed in any private premise above the age of five. In the United States, 31 states allow parents to provide alcohol to children and 19 permit consumption for a ‘family exception’. In other countries like Greece, alcohol laws do not apply at all in private premises. The assumption underpinning these laws is that alcohol consumption in supervised environments mitigates harm, and the findings of qualitative studies suggest parents share this view (Jones et al., 2016). However, the evidence on the risks associated with consumption with parents and in supervised environments is contentious.

The few studies investigating the association between consuming alcohol with parents and risky drinking have revealed highly discrepant findings. In a systematic review of seven prospective studies, parental supply of alcohol in childhood was associated with an increased odds of risky drinking later in adolescence (Sharmin et al., 2017). This review highlighted the diversity in assessments of parental supply – defined at home, with the family, under adult supervision, or supplied by parents – as a weakness of the literature. One subsequent prospective study found supply of alcohol from people other than parents was associated an increased risk of binge drinking and reporting symptoms of alcohol dependence (Mattick et al., 2018), but associations with specific people were not examined. None of these studies had an extended follow-up into early adulthood when alcohol use disorders have their peak age of onset (Degenhardt et al., 2016). The objective of this study was to examine the association between a number of social contexts of alcohol consumption and places of acquisition in adolescence, with the risk of high alcohol consumption and alcohol problems in early adulthood.

## 2 Methods

We used data from the 1970 British Cohort Study (Elliott and Shepherd, 2006), an ongoing longitudinal study of children born in Great Britain between the 5th and 11th April 1970. At 16 years of age, information was collected at a parental interview and participants completed self-report questionnaires. At 30 years, information was collected at participants home in a confidential interview. Written informed consent was given by parents of study participants.

A total of 16,571 babies born in England, Scotland and Wales were enrolled at birth and have been followed up on eight occasions across the life course. We used data on who alcohol was consumed with and source of acquisition at age 16 and outcomes at age 30 years. At the age of 16 (1986), of the 15,999 members traced and invited to participate, information was obtained from 11,615 (72.6%). At the age of 30 years, 14,087 members were traced and invited to participate, of whom 11,261 (68%) responded.

The two exposures of interest were where alcohol was consumed and acquired. At 16-years of age, the following questions were asked, “In the past 4 weeks, who have you had a drink with”, with options of a parent(s), a boy/girlfriend, other teenager(s), brother or sister, another adult(s), alone, or someone else. Participants were also asked, “If you drank alcohol since this time last week, where did you get it from?” with response options of their own home, a supermarket, off license, pub or bar, friend's home, relative's home, disco or party, or somewhere else. If participants did not list at least one place alcohol was consumed and one place it was acquired, they were not included in the sample. For both questions, participants were instructed to tick all people they had a drink with and sources alcohol was acquired. Using responses to these questions we calculated totals for the number of people participants drank with and sources alcohol was acquired.

The two alcohol outcomes as assessed at 30 years were: consuming >14 units of alcohol in the last week based on the UK Chief Medical Officers recommendation and screening positive for a possible alcohol problem using the CAGE questionnaire (questions included items on cutting down, being annoyed by criticism, feeling guilty, and eye-openers; Ewing, 1984). All participants, apart from lifelong teetotallers, were asked to complete questions on consumption of alcoholic beverages in the last week (from which units of alcohol were calculated using standard conversion criteria) and the CAGE questionnaire. A score of >2 on the CAGE questionnaire indicated a potential problem with alcohol (King, 1986).

Covariates were identified *a priori* due to an association with exposure or outcomes. At 16-years of age, these included the number of units of alcohol consumed in the past week, how frequently their mother and father consumed alcohol, and occupational social class based on mother's and father's occupation provided by parents at interview (Registrar General, 1980). At 30 years, participants achieved adults occupational social class was derived from their current occupation (Registrar General, 1980).

We imputed missing exposure and covariate data using multiple imputation by chained equations which included all variables in the prediction model to generate 20 datasets. We compared the characteristics of participants who did and did not provide complete data using Chi-square tests. We estimated the unadjusted phi correlation to measure the strength of correlation between exposures to assess the clustering of consumption in each social context with each source, using a linear regression model. Next, associations between exposures and outcomes were adjusted for sex, parental social class at 16 years, achieved adult social class at 30 years, the frequency of mothers and father's alcohol consumption, and the other people alcohol was consumed with (Model 1). We used linear regression to estimate the adjusted association between exposures with the number of units of alcohol consumed in the past week at 16 years of age. We used logistic regression to estimate adjusted associations between exposures and the two outcomes. We present these results as odds ratios (ORs) with accompanying 95% confidence intervals (CI). We found no evidence of interaction by sex between exposures and outcomes, so data were pooled. To examine potential mechanisms, we added the number of units of alcohol participants had consumed in the past week at 16 years of age to the model estimating associations between exposures and the two outcomes (Model 2). To examine the ‘shape’ of this association, we compared people with a score of zero to those who had consumed alcohol with 1 to ≥6 people. As the number of study members who drank with ≥6 people and those who acquired alcohol from ≥6 sources were < 1% scores above these levels were combined. The reference category in all analyses was not consuming alcohol in that social context or *via* that source. All models were then repeated with the sources in which alcohol had been acquired as the exposure. Sensitivity analyses were conducted after excluding participants with any missing data. All analyses were performed using Stata version 15.1. Our manuscript adheres to the guidelines for STrengthening the Reporting of OBservational studies in Epidemiology (von Elm et al., 2007).

## 3 Results

Of the 11,615 participants at 16 years of age, 10,968 (94.4%) took part in the resurvey at 30 years of age. Relative to study members without complete data, those with were more likely to have parents with a professional occupation, consume more units of alcohol in the last week, have parents who drank alcohol on most days, to drink with parents, other teens, brothers or sisters, other adults and acquire alcohol from a pub/bar, and their own home; and at 30-years consume >14 units in the last week and have a professional occupation. Table e1 (Supplementary file) shows 47.6% of participants were boys, most had parents with a skilled manual occupation, who drank alcohol occasionally, and participants consumed a mean of 7.37 (95% CI: 7.11, 7.64) units of alcohol in the past week at 16-years of age. By 30-years of age most worked in a skilled occupation.

**Fig. 1:**
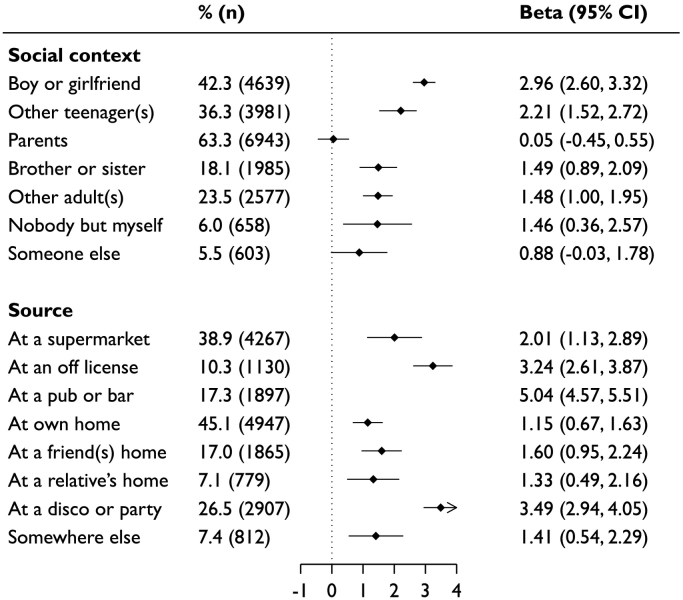
Coefficient (95% confidence interval) for association between social context of alcohol consumption and source with the units of alcohol consumed in the past week at 16-years of age (*n* = 10, 968) Adjusted for sex, parental social class at 16 years, achieved adult social class at 30 years, frequency of mothers alcohol consumption, frequency of fathers alcohol consumption, other social contexts alcohol is consumed in (other sources alcohol is acquired under source subheading). Reference category is not consuming in that context or source.

Of the 10,968 participants with a history of drinking alcohol, at 30-years of age, 32.1% consumed >14 units in the last week and 14.3% screened positive for a possible alcohol problem. Fig. 1 shows that, at 16 years of age, alcohol was most commonly consumed with other teenagers (63.3%) and sourced from a pub or bar (45.1%). The number of units consumed was lower when done so with parents than other contexts, and higher when alcohol was acquired from an off-license, pub or bar, disco or party than other sources (Fig. 1 and Table e2). The correlation heat map shown in Figure e1 shows there were few strong correlations between the social context and source. The upper left quadrant indicates consumption with boy/girlfriends and other teenagers was positively correlated with acquiring alcohol from a pub or bar, or disco or party, and consuming with other teenagers with acquisition from an off license. Consumption with parents was positively correlated with acquisition at home. Tables e3 and e4 show the percentage overlap between consumption in different social contexts and sources. The largest overlap in social contexts was found for those who consumed with other teenagers and for sources those who acquired alcohol from pubs or bars.

**Fig. 2:**
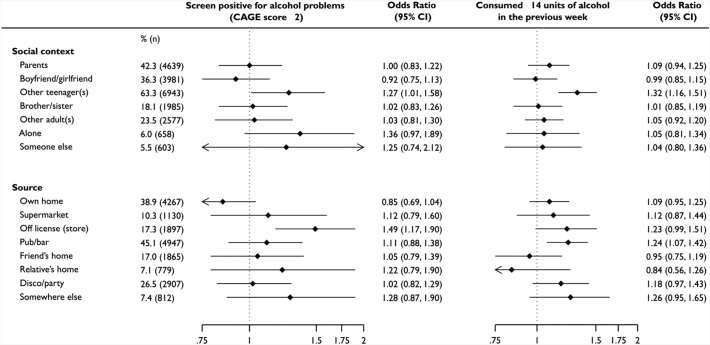
Odds ratio (95% confidence interval) for association between social context of alcohol consumption and source at 16-years with harmful alcohol consumption at 30-years of age (*n* = 10,968) Odds ratios adjusted for sex, parental social class at 16 years, achieved adult social class at 30 years, frequency of mothers alcohol consumption, frequency of fathers alcohol consumption, units consumed at 16 years of age, other social contexts alcohol is consumed in (other sources alcohol is acquired under source subheading). Note categories are not mutually exclusive so percentages do not sum to 100%. Reference category is not consuming in that context or source.

The sex-, socioeconomic status-, parent alcohol consumption-adjusted ORs for consuming alcohol with parents at 16 years was 1.09 (95% CI: 0.95, 1.25) for consuming >14 units in the last week, and 1.01 (95% CI: 0.84, 1.23) for having alcohol problems (Fig. 2). The adjusted ORs for alcohol acquired from participants own home was 1.10 (95% CI: 0.96, 1.27) for consuming >14 units in the last week and 0.86 (95% CI: 0.70, 1.06) for screening positive for possible alcohol problems. Consuming alcohol with other teenagers and acquisition from an off-license was associated with an increased risk of high consumption and alcohol problems. Adjustment for the number of units consumed at 16 years of age led to a modest attenuation of all ORs (Table e5). The odds of both outcomes increased in a dose-response fashion according to the number of people alcohol was consumed with and places it was acquired (Table e6).

Sensitivity analysis in the 1514 participants with no missing data in the analysis of alcohol problems and 1529 in the analysis of consuming >14 units at 30 years revealed essentially the same results (Table e7).

## 4 Discussion

In this longitudinal population-based sample, there was no suggestion that consuming alcohol with parents, or acquiring it at home, at 16 years of age, had a protective effect against high alcohol consumption or problems with alcohol at 30-years of age.

In the Family and Health study, drinking at home or outside the home at 13 to 16-years of age was related to alcohol problems 3-years later (van der Vorst et al., 2010). The Victorian Adolescent Health Cohort Study (VACS) investigators found that drinking with families and at pub/club(s), party(s) at 14–15 years of age was associated with an increased risk of consuming ≥5 units on one day in the past week by 15–17 years (Degenhardt et al., 2015). Our results suggest that drinking with parents was linked to lower levels of consumption at 16-years of age than when alcohol was consumed with other people. This did not, however, translate into a reduced or increased risk of later alcohol related harms in early adulthood. In contrast, we replicated findings of the VACS of an increased risk of later alcohol related harms associated with consumption at a pub or party. These inconsistencies may be because we separated parents from siblings, or that the risks of consuming with parents in adolescence do not extend to early adulthood.

One potential explanation for the associations we observed is that in adolescence consumption other teenagers and acquisition from off-license leads to more units being consumed. That associations remained after adjustment for the number of units consumed at 16 years of age provides little support for this hypothesis. An alternative hypothesis is that drinking with other teens, and acquisition from off-license in the mid-1980s was indicative of a greater propensity to binge drink than when acquired from other locations. This is consistent with evidence from The Second International Self-Report Delinquency Study (ISRD-2) that 57.6% of beer and wine and 68.2% of spirits were consumed with peers, and 23.5% beer and wine and no spirits were consumed with parents (Kask and Markina, 2014). This suggests that opportunities for parental monitoring of higher concentration alcohol is infrequent.

Our study has some advantages over previously published work. The 14-year follow-up meant the longevity of associations found in late adolescence could be tracked into adulthood. The main limitation of this study is loss to follow-up. This was mainly due to missing exposure data at 16-years when a national teachers strike reduced participation (Elliott and Shepherd, 2006), but sensitivity analyses provided no evidence that missing data introduced bias. That our assessment only focussed on consumption in the last week, not units consumed each day, meant that we could not examine associations with episodic heavy drinking. It is worthwhile clarifying that the questions used are on where alcohol was acquired not where it was consumed. We did not estimate interactions within or between contexts or sources in the estimating the risk of outcomes and only had a limited set of covariates available to us so that unmeasured confounding of the associations presented is likely. These findings may also not apply to Mediterranean countries where alcohol is often consumed in small amounts with meals and parents may have greater opportunity to model consumption within limits (Kuendig et al., 2008).

## 5 Conclusions

The implication that stem from our findings is that there was no clear reduction in the risk of excess alcohol consumption and alcohol-problems in early adulthood when alcohol was consumed with parents or at home in adolescence. This suggests that laws permitting consumption under 16 years of age with parents, or in private premises, such as adolescents' homes, may not minimise alcohol-related harm. That acquisition of alcohol from off-licenses was associated with high consumption and alcohol-problems lends some support to provisions in legislation that allow fines for underage sales and the initiatives that encourage the age verification before alcohol is purchased. As alcohol is a leading cause in car crashes, violence, and suicide among young people globally (Gore et al., 2011) the most sensible conclusion from the available evidence would seem to be that legislation and policy should recommend that alcohol is avoided, or at least consumption minimised, no matter who it is consumed with or where is it acquired.

## Funding

JW is funded by The Centre for the Development and Evaluation of Complex Interventions for Public Health Improvement (DECIPHer), a UKCRC Public Health Research Centre of Excellence. Joint funding (MR/KO232331/1) from the British Heart Foundation, Cancer Research UK, Economic and Social Research Council, Medical Research Council, the Welsh Government and the Wellcome Trust, under the auspices of the UK Clinical Research Collaboration, and is gratefully acknowledged. SB is supported by the National Institute for Health Research (NIHR) Blood and Transplant Research Unit in Donor Health and Genomics (NIHR BTRU-2014-10024), UK Medical Research Council (MR/L003120/1), British Heart Foundation (SP/09/002; RG/13/13/30194; RG/18/13/33946), and the NIHR Cambridge Biomedical Research Centre at the Cambridge University Hospitals NHS Foundation Trust. GDB receives finding from the UK Medical Research Council (MR/P023444/1) and the US National Institute on Aging (1R56AG052519-01; 1R01AG052519-01A1). These funders had no influence on the analysis, decision to publish, or preparation of this manuscript.

## Author contributions

JW designed the study. JW acquired the data and undertook the analyses. SB and GDB contributed to refining the analyses. All authors drafted the manuscript and approved its publication.

## Data availability

Data from the 1970 British Cohort Study is publicly available from the UK Data Archive. Further information on the procedures to obtain data from the 1970 British Cohort Study is described at: https://discover.ukdataservice.ac.uk/.

## Code availability

The code used to generate the results presented in the manuscript are available from the corresponding author on reasonable request.

## Declaration of competing interest

The authors declare they have no conflict of interest.

## Appendix A Supplementary data

Supplementary material Image 1

## References

[bb0005] Degenhardt L., Romaniuk H., Coffey C., Hall W.D., Swift W., Carlin J.B., O’Loughlin C., Patton G.C. (2015). Does the social context of early alcohol use affect risky drinking in adolescents? Prospective cohort study. BMC Public Health.

[bb0010] Degenhardt L., Stockings E., Patton G., Hall W.D., Lynskey M. (2016). The increasing global health priority of substance use in young people. Lancet Psychiatry.

[bb0015] Elliott J., Shepherd P. (2006). Cohort profile: 1970 British birth cohort (BCS70). Int. J. Epidemiol..

[bb0020] Ewing J.A. (1984). Detecting alcoholism. The CAGE questionnaire. JAMA.

[bb0025] General, R (1980). Classification of Occupations.

[bb0030] Gore F.M., Bloem P.J., Patton G.C., Ferguson J., Joseph V., Coffey C., Sawyer S.M., Mathers C.D. (2011). Global burden of disease in young people aged 10–24 years: a systematic analysis. Lancet.

[bb0035] Jones S.C., Andrews K., Berry N. (2016). Lost in translation: a focus group study of parents’ and adolescents’ interpretations of underage drinking and parental supply. BMC Public Health.

[bb0040] Kask K., Markina A. (2014). With whom did you drink last time? An analysis of Adolescents’ alcohol use. Annu. Res. Rev. Biol..

[bb0045] King M. (1986). At risk drinking among general practice attenders: validation of the CAGE questionnaire. Psychol. Med..

[bb0050] Kuendig H., Plant M.A., Plant M.L., Miller P., Kuntsche S., Gmel G. (2008). Alcohol-related adverse consequences: cross-cultural variations in attribution process among young adults. Eur. J. Pub. Health.

[bb0055] Mattick R.P., Clare P.J., Aiken A., Wadolowski M., Hutchinson D., Najman J., Slade T., Bruno R., McBride N., Kypri K., Vogl L., Degenhardt L. (2018). Association of parental supply of alcohol with adolescent drinking, alcohol-related harms, and alcohol use disorder symptoms: a prospective cohort study. Lancet Public Health.

[bb0060] NHS Digital (2017). Smoking, Drinking and Drug Use among Young People in England 2016.

[bb0065] Rehm J., Mathers C., Popova S., Thavorncharoensap M., Teerawattananon Y., Patra J. (2009). Global burden of disease and injury and economic cost attributable to alcohol use and alcohol-use disorders. Lancet.

[bb0070] Sharmin S., Kypri K., Khanam M., Wadolowski M., Bruno R., Mattick R.P. (2017). Parental supply of alcohol in childhood and risky drinking in adolescence: systematic review and meta-analysis. Int. J. Environ. Res. Public Health.

[bb0075] Steffens R., Sarrazin D. (2016). Guidance to reduce alcohol-related harm for young people. Background Paper. Münster: LWL-Coordination Office for Drug-Related.

[bb0080] van der Vorst H., Engels R.C.M.E., Burk W.J. (2010). Do parents and best friends influence the normative increase in adolescents’ alcohol use at home and outside the home?. J. Stud. Alcohol Drugs.

[bb0085] von Elm E., Altman D.G., Egger M., Pocock S.J., Gøtzsche P.C., Vandenbroucke J.P., STROBE Initiative (2007). Strengthening the reporting of observational studies in epidemiology (STROBE) statement: guidelines for reporting observational studies. BMJ.

