# Supplementary material for: Does the social context of early alcohol use affect alcohol-related harms in adulthood? Findings from a national birth cohort
Source: Prev Med. 2020 Jan;130:105947. doi: 10.1016/j.ypmed.2019.105947 (PMC6983927; doi:10.1016/j.ypmed.2019.105947)
Supplement: Supplementary file 1 — Supplementary material [file mmc1.docx]

**Table e1. Characteristics of participants 16-year and 30-years of age (n = 10, 968)**

|  | **N** | **%** |
| --- | --- | --- |
| **At 16-years of age** |  |  |
| Parents occupational social class ^a^ |  |  |
| Student | 268 | 2.44 |
| Unskilled | 302 | 2.75 |
| Partly skilled | 1062 | 9.68 |
| Skilled manual | 4316 | 39.35 |
| Skilled non-manual | 1172 | 10.69 |
| Managerial/ technical | 3077 | 28.05 |
| Professional | 772 | 7.04 |
| Frequency of fathers’ alcohol consumption |  |  |
| Never | 547 | 4.99 |
| Occasionally | 4254 | 38.79 |
| Rarely | 3729 | 34.00 |
| Some days | 1963 | 17.90 |
| Most days | 9 | 0.08 |
| Father not at home or dead | 465 | 4.24 |
| Frequency of mothers’ alcohol consumption |  |  |
| Never | 1289 | 11.75 |
| Occasionally | 5861 | 53.44 |
| Rarely | 2837 | 25.87 |
| Some days | 760 | 6.93 |
| Most days | 88 | 0.8 |
| Mother not at home or dead | 133 | 1.21 |
| **At 30-years of age** |  |  |
| Occupational social class ^a^ |  |  |
| Student | 8 | 0.07 |
| Unskilled | 241 | 2.20 |
| Partly skilled | 1227 | 11.19 |
| Skilled manual/ non-manual | 5012 | 45.7 |
| Managerial/ technical | 3788 | 34.54 |
| Professional | 691 | 6.30 |

^a^ Parents occupational social class: fathers response used unless missing and replaced with mothers occupation.

**Table e2. Units of alcohol consumed in past week at 16-years of age (n = 10, 968)**

|  | **Mean** | **95% confidence interval** |
| --- | --- | --- |
| **Social context** |  |  |
| Boy or girlfriend | 9.23 | 8.89, 9.57 |
| Other teenager(s) | 8.41 | 8.15, 8.67 |
| Parents | 7.63 | 7.30, 7.96 |
| Brother or sister | 9.08 | 8.54, 9.62 |
| Other adult(s) | 9.31 | 8.92, 9.70 |
| Nobody but myself | 9.72 | 8.59, 10.86 |
| Someone else | 7.72 | 6.70, 8.74 |
|  |  |  |
| **Source** |  |  |
| At a supermarket | 8.43 | 7.36, 9.50 |
| At an off license | 10.87 | 10.23, 11.51 |
| At a pub or bar | 10.21 | 9.86, 10.55 |
| At own home | 7.09 | 6.71, 7.46 |
| At a friend(s) home | 8.94 | 8.18, 9.71 |
| At a relative’s home | 7.95 | 6.68, 9.22 |
| At a disco or party | 10.14 | 9.60, 10.69 |
| Somewhere else | 6.77 | 5.76, 7.78 |

**
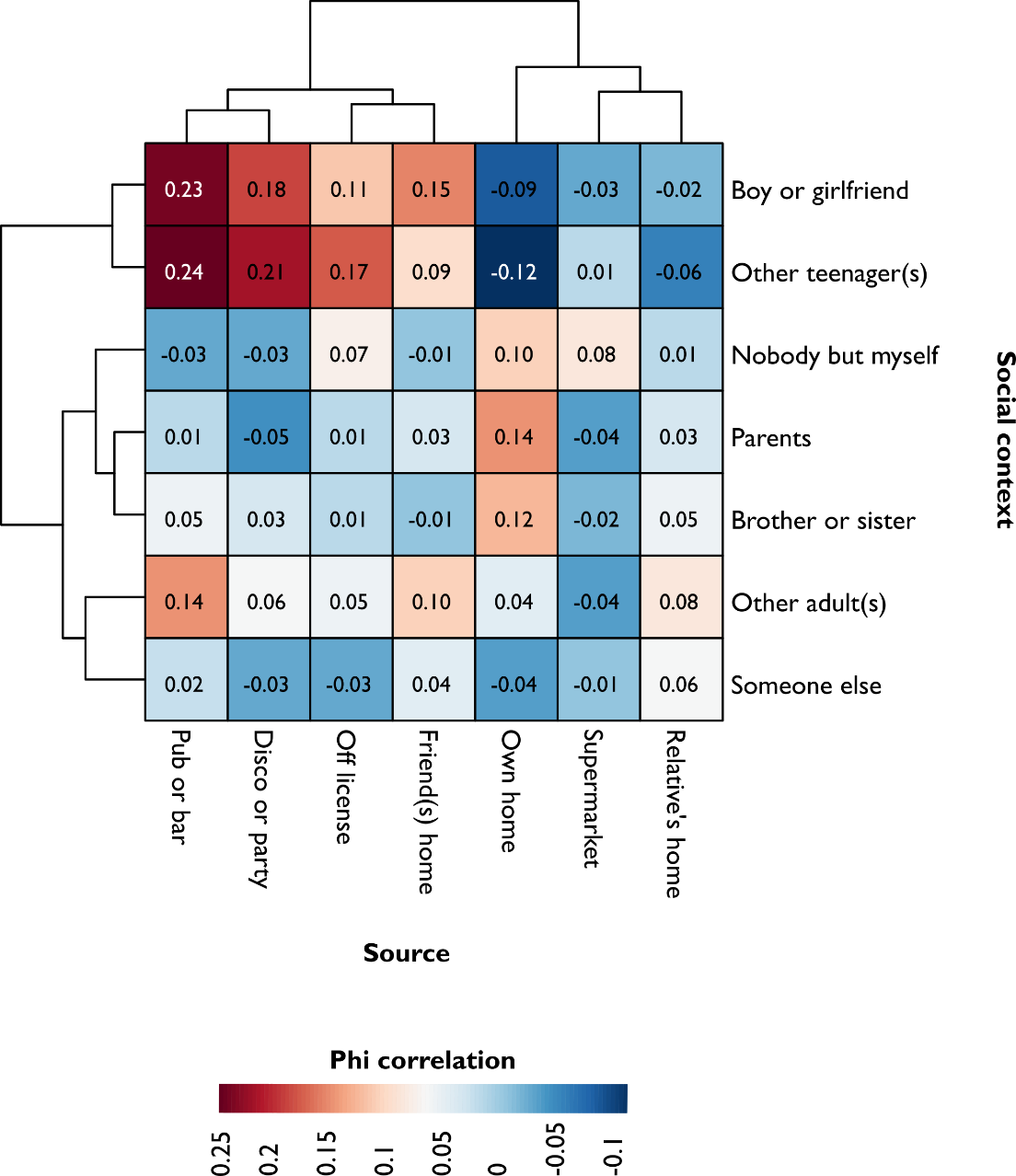
**

**Figure e1. Phi correlation coefficient heat map of the association between the social context of alcohol consumption and source at 16-years of age (n = 10, 968)**

**Table e3. Percentage overlap between specific social contexts for alcohol consumption (n = 10,968)**

| **Social context** | Parents | Boyfriend/girlfriend | Other teenager(s) | Brother/sister | Other adult(s) | Alone | Someone else |
| --- | --- | --- | --- | --- | --- | --- | --- |
| Parents | - | 37.19 | 63.62 | 29.29 | 31.27 | 6.46 | 4.05 |
| Boyfriend/girlfriend | 43.59 | - | 73.04 | 20.40 | 28.42 | 5.09 | 4.22 |
| Other teenager(s) | 42.96 | 42.08 | - | 20.23 | 26.86 | 6.16 | 3.71 |
| Brother/sister | 68.81 | 40.89 | 70.39 | - | 35.98 | 7.24 | 5.47 |
| Other adult(s) | 56.48 | 43.78 | 71.82 | 27.66 | - | 7.90 | 4.48 |
| Alone | 45.60 | 30.75 | 64.50 | 21.78 | 30.94 | - | 5.08 |
| Someone else | 31.25 | 27.79 | 42.47 | 17.96 | 19.18 | 5.54 | - |

**Table e4. Percentage overlap between specific sources of alcohol acquisition (n = 10,968)**

| **Source** | Own home | Supermarket | Off license (store) | Pub/bar | Friend’s home | Relative’s home | Disco/party | Somewhere else |
| --- | --- | --- | --- | --- | --- | --- | --- | --- |
| Own home | - | 7.78 | 14.11 | 33.19 | 18.24 | 8.41 | 19.76 | 4.73 |
| Supermarket | 29.24 | - | 12.64 | 28.96 | 14.09 | 7.98 | 24.13 | 6.27 |
| Off license | 31.78 | 12.64 | - | 47.74 | 22.31 | 5.47 | 28.57 | 3.90 |
| Pub/bar | 28.60 | 6.60 | 18.27 | - | 15.75 | 5.40 | 32.96 | 5.04 |
| Friend’s home | 42.75 | 8.78 | 23.19 | 42.84 | - | 10.04 | 33.01 | 3.74 |
| Relative’s home | 45.97 | 5.57 | 13.29 | 34.26 | 23.45 | - | 25.03 | 4.68 |
| Disco/party | 29.00 | 9.42 | 18.63 | 56.14 | 20.69 | 6.72 | - | 4.19 |
| Somewhere else | 24.91 | 6.27 | 9.14 | 30.93 | 8.47 | 4.53 | 15.07 | - |

**Table e5. Odds ratio (95 % confidence interval) for association between social context of alcohol consumption and source at 16-years with harmful alcohol consumption at 30-years of age (n = 10,968)**

|  | **Screen positive for alcohol dependency (CAGE score ≥2)** ^a^ | | **Consumed > 14 units alcohol in last week** ^a^ | |
| --- | --- | --- | --- | --- |
|  | **Model 1** | **Model 2** | **Model 1** | **Model 2** |
| **Social context** |  |  |  |  |
| Parents | 1.01 (0.84, 1.23) | 1.00 (0.83, 1.22) | 1.09 (0.95, 1.25) | 1.09 (0.94, 1.25) |
| Boyfriend/girlfriend | 0.98 (0.80, 1.19) | 0.92 (0.75, 1.13) | 1.04 (0.89, 1.21) | 0.99 (0.85, 1.15) |
| Other teenager(s) | 1.32 (1.06, 1.64) | 1.27 (1.01, 1.58) | 1.37 (1.20, 1.58) | 1.32 (1.16, 1.51) |
| Brother/sister | 1.06 (0.86, 1.30) | 1.02 (0.83, 1.26) | 1.03 (0.87, 1.22) | 1.01 (0.85, 1.19) |
| Other adult(s) | 1.06 (0.84, 1.34) | 1.03 (0.81, 1.30) | 1.08 (0.94, 1.23) | 1.05 (0.92, 1.20) |
| Alone | 1.39 (1.00, 1.94) | 1.36 (0.97, 1.89) | 1.07 (0.84, 1.37) | 1.05 (0.81, 1.34) |
| Someone else | 1.27 (0.75, 2.15) | 1.25 (0.74, 2.12) | 1.06 (0.81, 1.39) | 1.04 (0.80, 1.36) |
|  |  |  |  |  |
| **Source** |  |  |  |  |
| Own home | 0.86 (0.70, 1.06) | 0.85 (0.69, 1.04) | 1.10 (0.96, 1.27) | 1.09 (0.95, 1.25) |
| Supermarket | 1.16 (0.82, 1.63) | 1.12 (0.79, 1.60) | 1.14 (0.89, 1.46) | 1.12 (0.87, 1.44) |
| Off license (store) | 1.56 (1.21, 2.01) | 1.49 (1.17, 1.90) | 1.27 (1.04, 1.56) | 1.23 (0.99, 1.51) |
| Pub/bar | 1.18 (0.95, 1.47) | 1.11 (0.88, 1.38) | 1.31 (1.13, 1.51) | 1.24 (1.07, 1.42) |
| Friend’s home | 1.07 (0.81, 1.42) | 1.05 (0.79, 1.39) | 0.96 (0.77, 1.21) | 0.95 (0.75, 1.19) |
| Relative’s home | 1.24 (0.80, 1.93) | 1.22 (0.79, 1.90) | 0.84 (0.56, 1.28) | 0.84 (0.56, 1.26) |
| Disco/party | 1.08 (0.87, 1.33) | 1.02 (0.82, 1.29) | 1.22 (1.02, 1.47) | 1.18 (0.97, 1.43) |
| Somewhere else | 1.31 (0.88, 1.94) | 1.28 (0.87, 1.90) | 1.28 (0.97, 1.69) | 1.26 (0.95, 1.65) |

^a^ Model 1 adjusted for sex, parental social class at 16 years, achieved adult social class at 30 years, frequency of mothers alcohol consumption, frequency of fathers alcohol consumption, other social contexts alcohol is consumed in (other sources alcohol is acquired under source subheading); Model 2 is model 1 plus units consumed at 16 years of age. Note categories are not mutually exclusive so percentages do not sum to 100%. Reference category is not consuming in that context or source.

**Table e6. Odds ratio (95 % confidence interval) for association between the total score for the number of people alcohol is consumed with and sources it is acquired (n = 10, 968)**

|  |  | **Screen positive for alcohol dependency (CAGE score ≥2)** ^a^ | | **Consumed > 14 units alcohol in last week** ^a^ | |
| --- | --- | --- | --- | --- | --- |
|  | **%** | **Model 1** | **Model 2** | **Model 1** | **Model 2** |
| **Number of contexts alcohol consumed** |  |  |  |  |  |
| 0 | 10.9 | Reference | Reference | Reference | Reference |
| 1 | 27.8 | 1.05 (0.78, 1.43) | 1.04 (0.77, 1.41) | 1.29 (1.05, 1.59) | 1.27 (1.04, 1.57) |
| 2 | 30.6 | 1.22 (0.89, 1.68) | 1.16 (0.84, 1.60) | 1.41 (1.14, 1.76) | 1.36 (1.10, 1.68) |
| 3 | 19.6 | 1.29 (0.91, 1.81) | 1.19 (0.84, 1.68) | 1.53 (1.19, 1.95) | 1.42 (1.11, 1.81) |
| 4 | 8.3 | 1.35 (0.89, 2.04) | 1.20 (0.78, 1.85) | 1.66 (1.27, 2.16) | 1.49 (1.15, 1.94) |
| 5 | 2.5 | 1.58 (0.96, 2.58) | 1.36 (0.82, 2.25) | 1.84 (1.24, 2.74) | 1.61 (1.08, 2.40) |
| ≥6 | 0.3 | 2.39 (0.90, 6.34) | 1.93 (0.72, 5.18) | 2.07 (0.84, 5.07) | 1.71 (0.68, 4.25) |
| P for trend |  | 0.008 | 0.09 | <0.001 | 0.003 |
|  |  |  |  |  |  |
| **Number of sources alcohol acquired** |  |  |  |  |  |
| 0 | 7.4 | Reference | Reference | Reference | Reference |
| 1 | 40.7 | 1.04 (0.72, 1.51) | 1.00 (0.70, 1.46) | 1.23 (0.95, 1.58) | 1.19 (0.92, 1.53) |
| 2 | 32.7 | 1.19 (0.83, 1.72) | 1.10 (0.77, 1.55) | 1.43 (1.07, 1.91) | 1.32 (0.98, 1.78) |
| 3 | 14.6 | 1.48 (0.98, 2.23) | 1.29 (0.87, 1.92) | 1.62 (1.16, 2.25) | 1.43 (1.02, 2.01) |
| 4 | 3.7 | 1.78 (1.09, 2.89) | 1.48 (0.88, 2.51) | 1.82 (1.21, 2.72) | 1.53 (0.99, 2.35) |
| 5 | 0.7 | 1.29 (0.50, 3.32) | 1.03 (0.39, 2.69) | 2.09 (1.05, 4.16) | 1.69 (0.82, 3.48) |
| ≥6 | 0.09 | 1.75 (0.35, 8.53) | 1.28 (0.25, 6.59) | 2.08 (0.50, 8.67) | 1.58 (0.37, 6.81) |
| P for trend |  | <0.001 | 0.03 | <0.001 | 0.03 |

^a^ Model 1 adjusted for sex, parental social class at 16 years, achieved adult social class at 30 years, frequency of mothers alcohol consumption, frequency of fathers alcohol consumption; Model 2 is model 1 plus units consumed at 16 years of age. Reference category is not consuming in that context or source.

**Table e7. Odds ratio (95 % confidence interval) for association between social context of alcohol consumption and source at 16-years with harmful alcohol consumption at 30-years of age with no missing data**

|  | **Screen positive for alcohol dependency (CAGE score ≥2)** ^a^ **(n = 1,514)** | | **Consumed > 14 units alcohol in last week** ^a^  **(n = 1,529)** | |
| --- | --- | --- | --- | --- |
|  | **Model 1** | **Model 2** | **Model 1** | **Model 2** |
| **Social context** |  |  |  |  |
| Boy or girlfriend | 0.90 (0.65, 1.23) | 0.90 (0.65, 1.25) | 1.02 (0.81, 1.29) | 0.97 (0.76, 1.23) |
| Other teenager(s) | 1.25 (0.87, 1.79) | 1.25 (0.87, 1.81) | 1.17 (0.90, 1.52) | 1.12 (0.86, 1.46) |
| Parents | 1.05 (0.77, 1.44) | 1.05 (0.77, 1.45) | 1.16 (0.91, 1.46) | 1.15 (0.91, 1.45) |
| Brother or sister | 1.22 (0.86, 1.74) | 1.22 (0.86, 1.75) | 1.00 (0.76, 1.32) | 0.98 (0.75, 1.30) |
| Other adult(s) | 1.20 (0.86, 1.66) | 1.20 (0.86, 1.67) | 1.11 (0.86, 1.43) | 1.09 (0.85, 1.41) |
| Nobody but myself | 1.09 (0.63, 1.89) | 1.10 (0.63, 1.90) | 0.87 (0.56, 1.34) | 0.84 (0.54, 1.31) |
| Someone else | 0.98 (0.49, 1.95) | 0.98 (0.49, 1.95) | 0.95 (0.58, 1.58) | 0.92 (0.56, 1.53) |
|  |  |  |  |  |
| **Source** |  |  |  |  |
| At a supermarket | 0.88 (0.52, 1.49) | 0.89 (0.52, 1.51) | 0.91 (0.61, 1.35) | 0.89 (0.60, 1.32) |
| At an off license | 1.54 (1.07, 2.23) | 1.56 (1.07, 2.27) | 1.08 (0.80, 1.46) | 1.05 (0.77, 1.42) |
| At a pub or bar | 1.03 (0.76, 1.41) | 1.04 (0.75, 1.44) | 1.11 (0.88, 1.40) | 1.07 (0.84, 1.36) |
| At own home | 0.86 (0.70, 1.06) | 0.87 (0.63, 1.19) | 1.07 (0.85, 1.36) | 1.06 (0.84, 1.35) |
| At a friend(s) home | 1.08 (0.73, 1.59) | 1.09 (0.74, 1.61) | 0.97 (0.72, 1.30) | 0.95 (0.71, 1.29) |
| At a relative’s home | 1.59 (0.85, 2.98) | 1.60 (0.85, 3.00) | 0.74 (0.43, 1.27) | 0.73 (0.43, 1.25) |
| At a disco or party | 0.91 (0.64, 1.29) | 0.92 (0.64, 1.33) | 1.44 (1.12, 1.86) | 1.40 (1.07, 1.82) |
| Somewhere else | 1.15 (0.64, 2.04) | 1.15 (0.65, 2.06) | 1.12 (0.71, 1.75) | 1.10 (0.70, 1.73) |

^a^ Model 1 adjusted for sex, parental social class at 16 years, achieved adult social class at 30 years, frequency of mothers alcohol consumption, frequency of fathers alcohol consumption, other social contexts alcohol is consumed in (other sources alcohol is acquired under source subheading); Model 2 is model 1 plus units consumed at 16 years of age. Reference category is not consuming in that context or source.
